# A four-way, double-blind, randomized, placebo controlled study to determine the efficacy and speed of azelastine nasal spray, versus loratadine, and cetirizine in adult subjects with allergen-induced seasonal allergic rhinitis

**DOI:** 10.1186/1710-1492-9-16

**Published:** 2013-05-01

**Authors:** Anne K Ellis, Yifei Zhu, Lisa M Steacy, Terry Walker, James H Day

**Affiliations:** 1 Division of Allergy & Immunology, Department of Medicine, Queen’s University, Kingston, ON, Canada; 2 Allergy Research Unit, Kingston General Hospital, Kingston, ON, Canada; 3 Life Sciences, Queen’s University, Kingston, ON, Canada

**Keywords:** Allergic rhinitis, Azelastine, Environmental exposure unit, Onset of action, Cetirizine, Loratadine

## Abstract

**Background:**

Azelastine has been shown to be effective against seasonal allergic rhinitis (SAR). The Environmental Exposure Unit (EEU) is a validated model of experimental SAR. The objective of this double-blind, four-way crossover study was to evaluate the onset of action of azelastine nasal spray, versus the oral antihistamines loratadine 10 mg and cetirizine 10 mg in the relief of the symptoms of SAR.

**Methods:**

70 participants, aged 18-65, were randomized to receive azelastine nasal spray, cetirizine, loratadine, or placebo after controlled ragweed pollen exposure in the EEU. Symptoms were evaluated using the total nasal symptom score (TNSS). The primary efficacy parameter was the onset of action as measured by the change from baseline in TNSS.

**Results:**

Azelastine displayed a statistically significant improvement in TNSS compared with placebo at all time points from 15 minutes through 6 hours post dose. Azelastine, cetirizine, and loratadine reduced TNSS compared to placebo with an onset of action of 15 (p < 0.001), 60 (p = 0.015), and 75 (p = 0.034) minutes, respectively. The overall assessment of efficacy was rated as good or very good by 46% of the participants for azelastine, 51% of the participants for cetirizine, and 30% of the participants for loratadine compared to 18% of the participants for placebo.

**Conclusions:**

Azelastine’s onset of action for symptom relief was faster than that of cetirizine and loratadine. The overall participant satisfaction in treatment with azelastine is comparable to cetirizine and statistically superior to loratadine. These results suggest that azelastine may be preferential to oral antihistamines for the rapid relief of SAR symptoms.

## Introduction

Seasonal allergic rhinitis (SAR) is an inflammatory disease characterized by multiple symptoms including sneezing, rhinnorhea, nasal congestion, nasal and nasopharyngeal itching, and has associated ocular symptoms such as itchy, watery and red/burning eyes [1]. Oral antihistamines are often the first line treatment administered for SAR [2]. However, as SAR symptoms result from an interaction between inhaled allergens and IgE antibodies on mast cells located in the upper airway [3], it may be possible to achieve faster symptom relief through direct local delivery of a medication to the nasal tissues.

Azelastine is a second generation H_1_-antihistamine [4] that is currently marketed as a topically applied agent (i.e. nasal spray). Numerous studies have demonstrated its ability to provide significant improvement in the symptoms of SAR compared to placebo [5-9]. Azelastine is believed to exert its effects through alteration of the activities of mast cells, eosinophils, and neutrophils and inhibition of the synthesis or expression of leukotrienes, kinins, cytokines, and chemokines [10-13].

The regional and chronological fluctuations associated with the natural exposure to aeroallergens give rise to considerable inter-study variations when assessing the efficacy and onset of action of various drugs to treat SAR; therefore, this study was conducted in the highly controlled environment of the Environmental Exposure Unit (EEU). The EEU is a well-validated and internationally recognized controlled allergen challenge facility located in Kingston, ON Canada [14-16]. The EEU allows for large groups of clinical trial participants to be simultaneously exposed to controlled levels of airborne allergens such as ragweed or grass pollen. Within this specially designed room, allergen levels can be precisely maintained at predetermined levels and environmental variables such as air quality, temperature, humidity and CO_2_ levels are tightly regulated [15]. With the ability to control these variables, study conditions can be reproduced on different days at any time of the year with the same or different study participants, something that cannot be achieved with any other research model for allergic rhinitis. Utilizing this model thus yields more precise results for direct comparisons of different treatment modalities [14]. Over the past decade, the EEU has gained international acceptance for the clinical research conducted in Kingston with over 20 publications in top research journals (recent references indicated) [17-23].

Azelastine hydrochloride has been marketed as a prescription product in the United States since 1996 under the trade name Astelin®. A new dosing regimen of 1 spray per nostril twice daily was approved in 2006 for the treatment of SAR [24] and thus was administered in this study.

The objective of the current evaluation was to determine the onset of action of azelastine nasal spray, compared to established oral antihistamines (loratadine 10 mg and cetirizine 10 mg tablets), for the relief of symptoms of SAR. This study further allowed for the comparison of topical versus oral application of medication.

## Methods

### Study participants

Participants were healthy male and female volunteers between the ages of 18 and 65 with a history of SAR to ragweed for the preceding two consecutive pollen seasons. Atopic status was confirmed with a positive response to a skin prick test to ragweed allergen at screening or within 12 months of the screening visit (defined as a wheal diameter greater than or equal to 3 mm larger than the diluent control).

Enrolled female participants of childbearing potential used a medically acceptable form of birth control for at least 1 month prior to screening. Those who were not sexually active consented to use a double-barrier method should they become sexually active during the study. Females who were pregnant, lactating or had the intention of becoming pregnant were not enrolled.

Participants with a history of hypersensitivity to azelastine, loratadine, or cetirizine or were known to be nonresponsive to antihistamines were excluded. Participants with relevant concomitant disease (chronic sinusitis) or nasal structural abnormalities causing greater than 50% obstruction were also excluded. Furthermore, participants who suffered from an acute illness that could have interfered with the conduct of the study within 7 days of any pollen exposure visit were excluded. Also excluded were participants with asthma who required more than occasional use (<3 times per week) of inhaled short-acting β-2 agonists and any participants who took restricted medications within the proscribed time period prior to their first priming visit (See Table 1).

**Table 1:** Restricted medications and required washout times

**Prohibited medication**	**Time frame prohibited prior to first priming visit and thereafter**
Decongestants	Within 48 hours
Topical glucocorticoids	Within 14 days
Tricyclic antidepressants	Within 14 days
Tranquilizers	Within 14 days
Monoamine oxidase inhibitors	Within 14 days
Long acting β-2 agonists	Within 14 days
Glucocorticoids (inhaled, oral or intravenous)	Within 28 days
Glucocorticoids (intramuscular or intra-articular)	Within 84 days
Antihistamines	Within 7 days
Leukotrienes antagonists	Within 7 days
Theophylline	Within 7 days
Non-steroidal anti-inflammatory drugs	Within 7 days
Systemic antibodies	Within 7 days

Participants with clinically significant histories of hematological, renal, endocrine, pulmonary, gastrointestinal, cardiovascular, hepatic, psychiatric, or neurologic malignancies within the last 5 years were excluded. Other exclusion criteria include alcoholism or drug abuse within 2 years prior to the screening visit; regular use within 6 months of any type of tobacco product(s) or any smoking cessation nicotine-containing product; participation in any other trials involving investigational or marketed products within 30 days prior to the screening visit; and history of a positive test for HIV, TB (not due to vaccination), hepatitis B (not due to vaccination), or hepatitis C.

### Study design

This Phase IV trial was a randomized, single-center, double-blind, placebo-controlled, double-dummy, four-way crossover study. All participants provided written, informed consent prior to study entry. The trial protocol, amendments and informed consent forms were approved by the Queen’s University Health Sciences and Affiliated Teaching Hospitals Research Ethics Board, and the study was conducted according to Good Clinical Practice standards and International Conference on Harmonization guidelines.

The study was conducted in the Environmental Exposure Unit (EEU) and consisted of a screening visit, a priming period and four dosing/exposure periods with a 13-day washout between each period.

Eligibility was determined at the screening visit, during which written informed consent was obtained. The first priming visit occurred within 16 days of the screening visit. Participants attended a minimum of one up to a maximum of five priming visits, where they were exposed to ragweed pollen in the EEU to establish an adequate level of allergic reactivity. Participants underwent up to 3 hours of pollen exposure at each visit, during which symptoms were recorded on diary cards calculating the Total Nasal Symptom Score (TNSS) every 30 minutes.

The TNSS was comprised of the following symptoms of allergic rhinitis: sneezing, runny nose, and itchy nose, with each individual symptom rated on a 4-point scale (0 = none, 1 = mild, 2 = moderate, 3 = severe; See Table 2). Thus, the maximum TNSS that could be achieved was 9. Also documented were symptom score ratings for nasal obstruction, itchy eyes and teary eyes.

**Table 2:** Symptoms score definitions

**Score**	**Grade**	**Guideline**
0	None	No sign/symptom is evident
1	Mild	Sign/symptom clearly present, but minimal awareness; easily tolerated
2	Moderate	Definite awareness of sign/symptom that is bothersome, but tolerable
3	Severe	Sign/symptom that is hard to tolerate; causes interference with activities during the challenge session

A minimum TNSS of 4 must have been obtained at the 90 minute symptom evaluation during a priming visit; those who did not meet this criterion were asked to return for another priming visit up to a maximum of five visits. Participants who met this criterion during at least one priming visit returned within 7 days for the first of four dosing periods.

**Figure 1:**
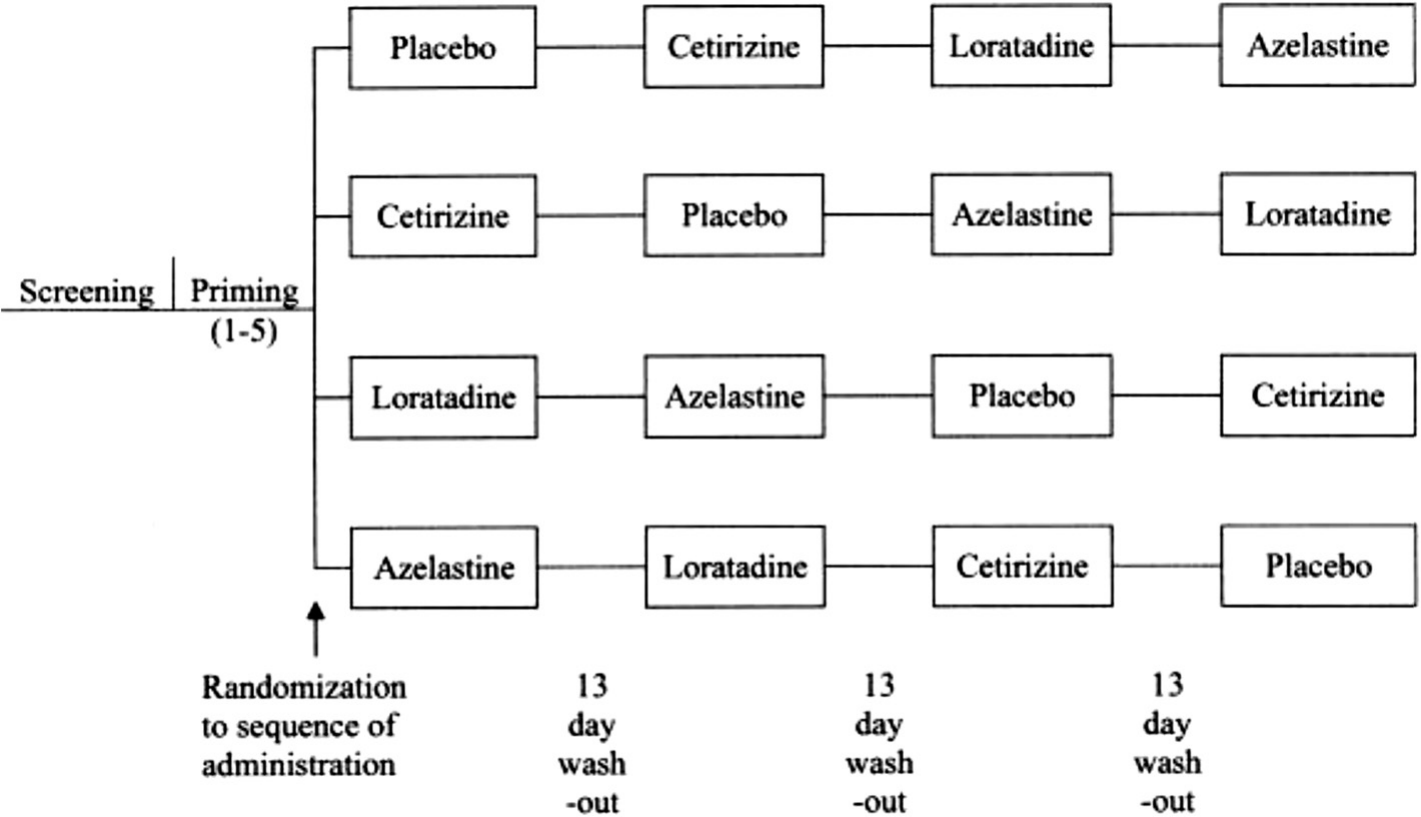
Study diagram.

Each dosing period consisted of an 8 hour allergen challenge. Participants were asked to score symptoms on diary cards every 30 minutes during a 2 hour baseline allergen challenge period. At 90 minutes, the participant must have had a minimum TNSS of 4 in order to be randomized into the study. Participants were randomized to the sequence of administration of one dose of each of the four study medications – azelastine (A), loratadine (L), cetirizine (C), or placebo (P). Randomization occurred in a 1:1:1:1 ratio, with approximately 17 participants randomized to each of the treatment sequences (Figure 1).

At 2 hours, participants were administered their assigned treatment, receiving oral medication with placebo nasal spray, nasal medication with placebo tablet, or placebo nasal spray and placebo tablet as control. Following dosing, the allergen challenge continued for 6 hours and participants were asked to score symptoms on diary cards every 15 minutes for the first 2 hours and every 30 minutes for the remaining 4 hours. Participants also completed an overall assessment of treatment efficacy diary card. Lastly, participants were questioned at the end of each dosing period with regards to the occurrence of adverse events.

### Statistical analysis

The Per Protocol (PP) population consisted of all participants who completed all four dosing periods. A priori, it was established that data from these participants were used for the primary comparison of the four treatment groups. The Intent-To-Treat (ITT) population consisted of participants who provided at least one estimate of an efficacy parameter after the first dose of study treatment and this data was used as support in the estimation of the onset of action and efficacy of the four treatments.

Absolute values and change from baseline were summarized for TNSS, the individual component symptoms (sneezing, runny nose, and itchy nose), average TNSS over the last 2 hours, nasal obstruction, teary eyes, and itchy eyes. The data were described by summary statistics. Mean TNSS, sneezing, runny nose, itchy nose, stuffy nose, teary eyes, and itchy eyes and the corresponding mean change from the baseline were plotted across time.

For each time point, mean change from baseline for azelastine, cetirizine, and loratadine was compared to mean change from baseline for placebo. Corresponding 95% confidence intervals (CI) were presented. Differences between mean change from baseline and corresponding 95% CI were also presented for treatment differences between azelastine and cetirizine and between azelastine and loratadine. For continuous variables, estimates and p-values were obtained from a mixed effects model with fixed effects for sequence, period and treatment and random effects for participant within sequence. Statistical tests were performed at a nominal two-sided level of P = 0.05. No adjustments for multiplicity were made. For overall assessment of efficacy, estimates and p-values were obtained from a mixed effects cumulative logit proportional odds model with fixed effects for sequence, period and treatment and random effects for participant within sequence. All statistical analyses were performed using SAS® software, version 9.1.

## Results

A total of 70 participants were randomized and all participants took at least one dose of study drug and thus received at least one efficacy evaluation. All 70 participants were included in the ITT population; however 4 participants were excluded from the PP population for failing to complete all four dosing periods or for lacking the required symptom score. The demographic characteristics of study participants and the baseline symptom scores prior to dosing period 1 are summarized in Table 3 and were similar among the four treatment sequences.

**Table 3:** Participants’ baseline demographic characteristics and symptom scores

**Variable**	**Overall (N = 66)**	**Variable**	**Overall (N = 66)**
**Age (yrs)**		**Baseline sneezing score**	
Mean	35.0	Mean	2.1
Std.	9.88	Std.	0.97
Median	34.5	Median	2.0
Min. to Max.	21-63	Min. to Max.	0-3
**Gender**		**Baseline runny nose score**	
Male	27 (41%)	Mean	2.7
Female	39 (59%)	Std.	0.44
**Ethnicity**		Median	3.0
Hispanic	0 (0%)	Min. to Max.	2-3
Not Hispanic	66 (100%)	**Baseline nasal itching score**	
**Race**		Mean	2.6
Caucasian	64 (97%)	Std.	0.55
Black	0 (0%)	Median	3.0
Asian	2 (3%)	Min. to Max.	1-3
American Indian/Alaska Native	0 (0%)	**Baseline stuffy nose score**	
Native Hawaiian/Other Pacific Islander	0 (0%)	Mean	2.6
Other	0 (0%)	Std.	0.52
**Height (cm)**		Median	3.0
Mean	168.2	Min. to Max.	1-3
Std.	8.70	**Baseline teary eyes score**	
Median	168.0	Mean	2.0
Min. to Max.	153-188	Std.	0.73
**Weight (kg)**		Median	2.0
Mean	79.6	Min. to Max.	1-3
Std.	16.77	**Baseline itchy eyes score**	
Median	79.0	Mean	2.4
Min. to Max.	50-125	Std.	0.75
**BMI (kg/m**^**2**^**)**		Median	3.0
Mean	28.11	Min. to Max.	1-3
Std.	5.478	**Baseline total nasal symptom score**	
Median	27.04	Mean	7.4
Min. to Max.	19.1-42.8	Std.	1.21
		Median	7.0
		Min. to Max.	5-9

Baseline is the 90-minute evaluation after the beginning of the allergen challenge for the baseline period prior to dosing period 1.

**Figure 2:**
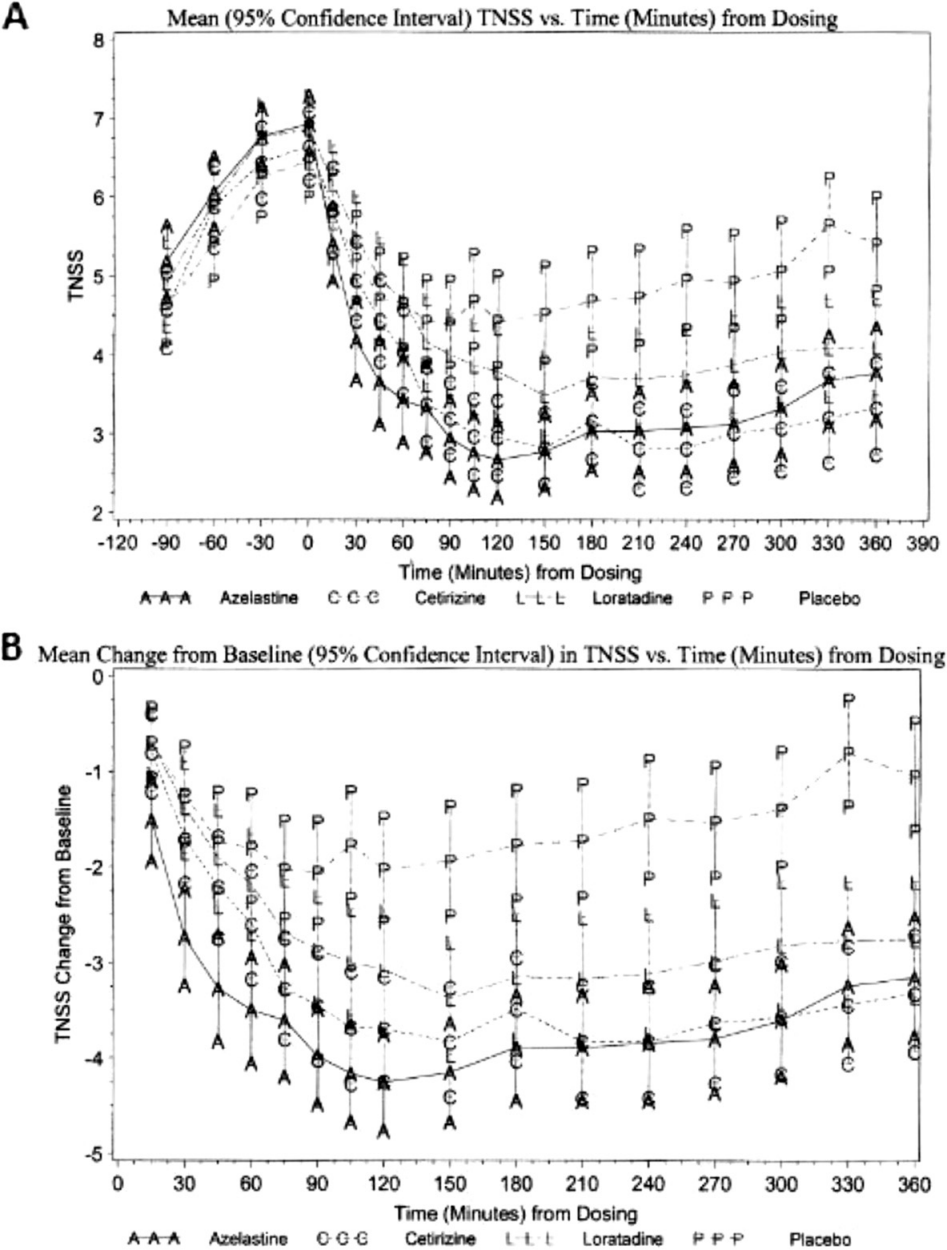
**Change in TNSS over time; comparison between Azelastine (AAA), Cetirizine (CCC) and Loratadine (LLL) vs. Placebo (PPP). A**) Mean (95% confidence interval) TNSS and **B**) Mean change from baseline (95% confidence interval) in TNSS vs. Time (minutes) from dosing.

The primary efficacy parameter was the onset of action measured by the change from baseline in TNSS. For each of the active treatment groups, onset of action was defined as the time after treatment when the drug demonstrated a statistically significant change that was maintained until the next consecutive time point compared to placebo. Azelastine showed a statistically significant improvement in the TNSS at 15 minutes compared with placebo (p < 0.001), and the effect was durable at each time point during the 6 hours post-dose (p < 0.001). Cetirizine and loratadine displayed a statistically significant improvement in the TNSS at 60 minutes (p = 0.015) and 75 minutes (p = 0.034), respectively, compared with placebo; the effect was durable at each time point thereafter through 6 hours post dose (p < 0.001 and p ≤ 0.011, respectively). The mean TNSS and mean change from baseline in TNSS for all three medications and placebo are shown in Figure 2.

Azelastine was more effective than cetirizine at each time point from 15 to 60 minutes post-dose (95%CI ≤ -0.2) and more effective than loratadine at each time point from 15 minutes to 5 hours post dose (95%CI ≤ -0.1). The raw mean changes from baseline in TNSS ranged from -0.7 (at 15 minutes) to -2.1 (at 90 minutes) for placebo, from -0.8 (at 15 minutes) to -3.8 (at 2.5, 3.5, and 4 hours) for cetirizine, from -0.7 (at 15 minutes) to -3.4 (at 2.5 hours) for loratadine, and from -1.5 (at 15 minutes) to -4.3 (at 120 minutes) for azelastine. The greater change of 0.7 in azelastine at 15 minutes post-dose in comparison to cetirizine indicates an immediate and clinically relevant increase in tolerability of symptoms; which would translate into decreased interference with daily functioning.

The secondary efficacy parameter was measured by four components: change from baseline for the individual components of the symptoms constituting the TNSS (sneezing, itchy nose, and runny nose); the average TNSS change from baseline over the last 2 hours of the allergen challenge; the relief of nasal obstruction, teary eyes, itchy eyes; and the overall participant assessment of efficacy.

**Figure 3:**
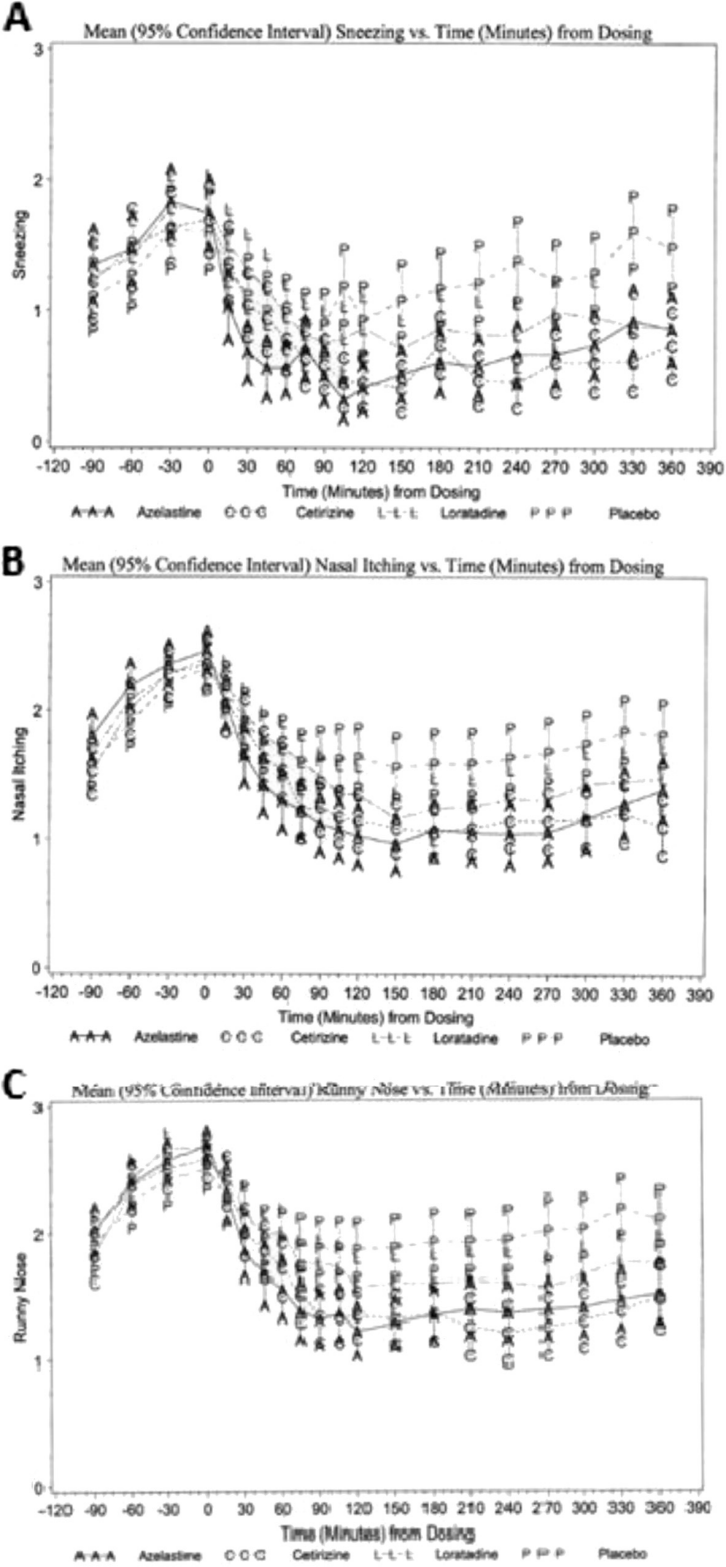
**Change in individual symptom components over time between Azelastine (AAA), Cetrizine (CCC) and Loratadine (LLL) vs. Placebo (PPP).** Mean (95% confidence interval) component score vs. Time from dosing for **A**) Sneezing **B**) Itchy nose **C**) Runny nose.

Figure 3 illustrates the mean component scores for each medication for sneezing, nasal itching and runny nose. Azelastine showed significant improvement in the sneezing score and the itchy nose score at 15 minutes compared with placebo (p = 0.007), and at 30 minutes for runny nose compared with placebo (p < 0.001). This effect was durable at each time point during the 6 hours post-dose (p ≤ 0.047, p < 0.001, and p < 0.001, respectively). Cetirizine showed significant improvement in the sneezing score and itchy nose score at 75 minutes compared with placebo (p = 0.026 and p < 0.001, respectively) and at 30 minutes for runny nose compared with placebo (p = 0.043). Loratadine showed statistically significant improvements in the sneezing score and itchy nose score at 105 minutes compared to placebo (p = 0.002 and p = 0.013, respectively) and at 75 minutes for the runny nose score compared with placebo (p = 0.016). The raw mean changes from baseline in sneezing score ranged from -0.3 (15 minutes) to -1.3 (2.5 hours) for cetirizine, from -0.3 (15 minutes) to -1.1 (2.5 hours) for loratadine, and from -0.7 (15 minutes) to -1.4 (105 minutes) for azelastine. The raw mean changes from baseline in itchy nose score ranged from -0.3 (15 minutes) to -1.3 (2.5, 3.0, 3.5, and 6.0 hours) for cetirizine, from -0.2 (15 minutes) to -1.2 (2.5, 3.0, and 3.5 hours) for loratadine, and from -0.4 (15 minutes) to -1.5 (2.5 hours) for azelastine. The raw mean changes from baseline in runny nose ranged from -0.2 (15 minutes) to -1.4 (4 hours) for cetirizine, from -0.2 (15 minutes) to -1.1 (120 minutes and 2.5, 3.0, 3.5, 4.0, and 4.5 hours) for loratadine, and from -0.4 (15 minutes) to -1.5 (120 minutes) for azelastine.

Azelastine was more effective than cetirizine at each time point from 15 to 45 minutes post-dose and more effective than loratadine at each time point from 15 to 60 minutes and 105 to 120 minutes post dose for the sneezing score. It was also more effective than cetirizine at each time point from 30 to 60 minutes post-dose and more effective than loratadine at each time point from 15 minutes to 5 hours post dose with the exception of the 3 hour time point for the itchy nose score. Azelastine was more effective than cetirizine at each time point from 15 to 60 minutes post-dose and more effective than loratadine at each time point from 30 minutes to 6 hours post dose with the exception of 4.5 hours for the runny nose score.

The change from baseline for azelastine, cetirizine, and loratadine were significantly different from the change from baseline for placebo (p < 0.001) for the average TNSS over the last 2 hours of the allergen challenge. No statistically significant differences were observed between azelastine and cetirizine (p = 0.866) nor between azelastine and loratadine (p = 0.066).

Azelastine showed a statistically significant improvement in the stuffy nose score and itchy eyes score at 15 minutes compared with placebo (p = 0.029, p = 0.028, respectively), and at 45 minutes in the teary eyes score compared with placebo (p = 0.002). The effect was durable for each time point in the 6 hours post dose for all three symptoms (p ≤ 0.029, p ≤ 0.006, and p ≤ 0.049, respectively), with the exception of at 75 minutes for the teary eyes score. Cetirizine showed statistically significant improvement in the stuffy nose score at 60 minutes (p = 0.029), in the itchy eyes score at 15 minutes (p = 0.039), and in the teary eyes score at 105 minutes (p = 0.001) compared with placebo. Loratadine showed a statistically significant improvement in the stuffy nose score at 3 hours (p < 0.001), and in the teary eyes score at 105 minutes (p = 0.005) compared with placebo. Loratadine showed a statistically significant improvement at 15 minutes and 45 minutes in the itchy eyes score compared with placebo (p = 0.028 and p = 0.033, respectively), the effect was durable at each time point at 75 minutes through 6 hours post-dose (p ≤ 0.016).

Azelastine was more effective than cetirizine at 15 minutes post-dose and more effective than loratadine at each time point from 15 to 60 minutes post dose except at the 30 minute time point for the stuffy nose score. Azelastine was also more effective than cetirizine and loratadine at 45 and 60 minutes post-dose for the itchy eyes score. No statistically significant differences were observed in relief of teary eyes symptoms between azelastine and cetirizine or loratadine at any time point.

Better overall assessment of efficacy was shown for azelastine, cetirizine, and loratadine compared to placebo (p < 0.001, p < 0.001, and p = 0.003, respectively). The overall assessment of efficacy was completed on a 4-point scale (1 = very good, 2 = good, 3 = satisfactory, 4 = insufficient). Of the 66 participants who completed all four dosing treatments, the overall assessment of efficacy was rated as very good or good by 30 participants for azelastine, 34 participants for cetirizine, and 20 participants for loratadine compared to 12 participants for placebo. Overall assessment of efficacy for azelastine was similar to cetirizine (p = 0.313) but significantly better than loratadine (p = 0.014). Detailed assessment of overall efficacy for all three drugs and placebo is shown in Table 4.

**Table 4:** Participant ratings of overall effectiveness of the medication

	**Placebo**	**Cetirizine**	**Loratadine**	**Azelastine**
	**(N = 66)**	**(N = 66)**	**(N = 66)**	**(N = 66)**
**Very good**	6 (9%)	14 (21%)	8 (12%)	11 (17%)
**Good**	6 (9%)	20 (30%)	12 (18%)	19 (29%)
**Satisfactory**	10 (15%)	20 (30%)	22 (33%)	21 (32%)
**Insufficient**	44 (67%)	12 (18%)	24 (36%)	15 (23%)
**Mean**	3.4	2.5	2.9	2.6
**Std.**	0.99	1.03	1.02	1.02
**Median**	4.0	2.0	3.0	3.0
**Min. to Max.**	1-4	1-4	1-4	1-4
**P value vs. placebo**		<0.001	0.003	<0.001

P-values and estimates obtained from a mixed effects cumulative logit proportional odds model with fixed effects for sequence, period, and treatment and random effects for subject within sequence.

Azelastine, cetirizine, and loratadine were well tolerated, and few adverse events were reported. For azelastine, all except 1 of the adverse events were mild or moderate in intensity, and all except 2 adverse events were considered not possibly related to the study medication. The severe adverse event was sinus headache, and the 2 possibly related adverse events were moderate somnolence and mild dysgeusia. The most commonly reported adverse event was myalgia (3 subjects), followed by headache (2 subjects), diarrhea (2 subjects), and nasal congestion (2 subjects). For cetirizine, all except 1 of the adverse events were mild or moderate in intensity, and all adverse events were considered not possibly related to the study medication by the investigator. The severe adverse event was abdominal pain. No adverse event was reported by more than 1 subject. For loratadine, all adverse events were mild or moderate in intensity, and all except 1 adverse event were considered not possibly related to the study medication. The possibly related adverse event was mild urticaria. The only adverse event reported by more than 1 subject was upper respiratory tract infection. For placebo, all adverse events were mild or moderate in intensity and considered not possibly related to study medication. No adverse event was reported by more than one subject. No participants elected to discontinue the study due to adverse events.

## Discussion

This study was designed to characterize the exact onset of action for allergic rhinitis symptom relief by azelastine (1 spray per nostril) compared to the onset of action of established oral antihistamines loratadine 10 mg and cetirizine 10 mg tablets.

Azelastine’s onset of action for TNSS, occurring at 15 minutes, was faster than the onset of action for cetirizine and loratadine. This rapid onset of action is consistent with previous environmental exposure facility trials [25,26], which also demonstrated an azelastine onset of action for TNSS of 15 minutes.

Azelastine demonstrated greater symptom score reduction than cetirizine during the immediate period post-dose and better efficacy than loratadine for the majority of the period post-dose (Figure 2). This suggests that azelastine may be preferential to oral antihistamines for the rapid relief of SAR symptoms. In vitro studies using rat IgE-producing hybridoma FE-3 cells have shown azelastine to have an inhibitory effect on IgE secretion [27]. While this has not been shown with human cells nor in vivo, it is possible that azelastine may confer rapid relief through inhibition of allergen-antibody interactions associated with SAR symptoms in the upper airway. Furthermore, the topical application of azelastine may allow for more rapid absorption in comparison to the orally taken cetirizine and loratadine, thereby accounting for its faster onset of action.

Azelastine’s onset of action for the relief of the individual components of TNSS (sneezing, itchy nose, and runny nose) was also faster than the onset of action for cetirizine and loratadine. Azelastine achieved durable significant improvement at 15 minutes for sneezing and itchy nose and at 30 minutes for runny nose. Cetirizine and loratadine did not achieve a durable significant response for all components until at least 60 minutes and 75 minutes post-dose, respectively. Overall, azelastine was able to decrease the TNSS component scores more quickly, and was able to maintain the decreased score at a level comparable to or better than cetirizine and loratadine over the ensuing 6 hours post-dose (Figure 3). It should be noted that the oral medications were as or almost as effective over the last 2 hours of the allergen challenge in treating TNSS. There were no statistically significant differences between the average TNSS change from baseline over the last two hours for all three medications. Thus, azelastine provided comparable relief of TNSS symptoms during the later period post-dose.

Azelastine showed a faster onset of action for the relief of stuffy nose and teary eyes than cetirizine and loratadine. Faster relief of stuffy nose is significant as nasal congestion has been reported as the most bothersome rhinitis symptom by more than half of 3206 patients surveyed with histories of rhinitis [28]. Azelastine and cetirizine both showed an onset of action of 15 minutes for the relief of itchy eyes, which was faster than the onset of action for loratadine. For the overall participant satisfaction in treatment, azelastine was comparable to cetirizine and statistically superior to loratadine (Table 4).

No safety concerns were identified in this study, with all active preparations being safe and well tolerated.

The effectiveness and onset of action of cetirizine 10 mg and loratadine 10 mg compared to placebo has previously been studied [29,30], with results that are consistent with the findings of this trial. Both studies found the onset of action for multi-component symptom scores to be approximately 1 hour for cetirizine and approximately 3 hours for loratadine. This is consistent with the current results as cetirizine’s onset of action occurred at approximately 1 hour for most symptoms evaluated. Loratadine’s onset of action occurred more quickly in this trial than in these previous trials; however, its onset of action was consistently longer than cetirizine and azelastine, not occurring until at least 75 minutes for all symptoms. One point to consider as well is that the double-dummy nature of these types of studies may lead to enhanced efficacy in the antihistamine arms due to the known therapeutic benefits derived from nasal saline (placebo) application that would be delivered to the oral antihistamine treated participants.

Other trials have examined azelastine (2 sprays per nostril) in comparison to cetirizine 10 mg for the treatment of seasonal allergic rhinitis [31,32]. These studies examined TNSS scores over the course of 14 days and therefore onset of action was not the main objective. Azelastine showed greater improvements in TNSS symptoms than cetirizine over the 14 days in both studies. A more appreciable difference in total TNSS may have been observed in this study had the maximum TNSS score been greater than 9.

Azelastine (2 sprays per nostril) has also been studied for its efficacy in conjunction with loratadine 10 mg [33]. The combination of azelastine and loratadine was compared to azelastine alone and desloratadine 5 mg. This study found azelastine to be an effective alternative for those with poor response to loratadine. However, the individual efficacies of azelastine and loratadine were not compared in this study.

## Conclusions

To our knowledge, this is the first trial directly comparing the onset of action of azelastine, cetirizine, and loratadine for the treatment of SAR. The unique operational characteristics of the EEU facilitated this head-to-head comparison. This study supports the rapid onset of action of azelastine nasal spray to relieve SAR symptoms; more quickly than oral antihistamines. The faster onset combined with comparable levels of symptom relief suggest that azelastine could be used as a replacement for oral antihistamines in the management of SAR.

## Abbreviations

ITT: Intent to treat; PP: Per protocol; SAR: Seasonal allergic rhinitis; TNSS: Total nasal symptom score

## Competing interests

Dr Ellis has received speaking honoraria from Pfizer, Merck and Sanofi, has served on an Advisory Board for Sanofi, and has received research grants from Circassia Ltd, Adiga Life Sciences, and GlaxoSmithKline in the last 12 months. All other authors declare that have no competing interests.

## Authors’ contributions

AKE was involved in drafting of the manuscript and critically revising its contents. YZ drafted the manuscript and assisted with subsequent revisions. LMS participated in subject recruitment and was a study coordinator for the study. TW ensured appropriate levels of ragweed pollen in EEU. JHD was involved in protocol revisions for the study and carried out the study for data procurement. All authors read and approved the final manuscript.

## Author’s information

James H. Day: Post-humous.
